# Accurate data as the foundation of equitable cancer control: Lessons from global cancer statistics 2024 and the complexity of ovarian cancer

**DOI:** 10.3322/caac.70096

**Published:** 2026-07-17

**Authors:** Christina R. Washington, Kathleen N. Moore, Isabelle L. Ray‐Coquard

**Affiliations:** ^1^ Stephenson Cancer Center University of Oklahoma Oklahoma City Oklahoma USA; ^2^ Fred & Pamela Buffett Cancer Center University of Nebraska Medical Center Omaha Nebraska USA; ^3^ Centre Léon Bérard University Claude Bernard Lyon I Lyon France

In this issue of *CA: A Cancer Journal for Clinicians*, Sung and colleagues present global cancer statistics 2024, the latest in the GLOBOCAN series produced by the International Agency for Research on Cancer.^1^ They estimate 20.6 million new cancer cases and 9.8 million cancer deaths worldwide in 2024, equivalent to one in five people developing cancer in their lifetime, and project that the annual burden will reach 31.5 million cases by 2050, a 53% increase driven largely by population growth and aging.^1^ The largest proportional increases will fall on low Human Development Index (HDI) and medium‐HDI countries, *where the number of new cases is projected to roughly double*. The value of these figures for setting screening, prevention, and treatment policy depends on the accuracy of the data used to generate them. This accuracy is weakest in parts of the world where the cancer burden is projected to grow fastest.

Sung and colleagues are explicit about this limitation. They note that only about one in three countries reports high‐quality incidence data to the International Agency for Research on Cancer's Cancer Incidence in Five Continents, whereas only one in four countries reports high‐quality national mortality data to the World Health Organization.^1^ Population‐based cancer registries (PBCRs) capture a small fraction of the population across much of sub‐Saharan Africa and Asia, and national vital statistics systems frequently suffer from incomplete coverage and from reliance on nonspecific causes of death that must be statistically redistributed.^2^, ^3^ Therefore, the 2024 estimates are best understood as a synthesis of observed data, short‐term prediction, and modeled mortality‐to‐incidence ratios, largely extrapolated from data collected before 2020.^1^ The robustness of that synthesis varies widely by region, and this variability can affect the global estimates on which policymakers, funders, and clinicians base their decisions.

These limitations have practical consequences. When the two principal global frameworks, GLOBOCAN and the Global Burden of Disease Study, disagree on basic questions such as which cancer is most commonly diagnosed worldwide, the discrepancy arises from differences in data processing, modeling strategy, and the redistribution of ill‐defined mortality codes rather than from differences in underlying biology.^1^, ^4^ Most registries also lack individual‐level data on socioeconomic status, a key surrogate for the social determinants that drive much of the variation in incidence and outcome, and this limits how meaningfully registry data can be translated into targeted intervention.

Global cancer statistics 2024 illustrates why data annotation beyond only incidence and mortality matters. The report documents a marked decoupling of incidence and mortality by HDI. Prostate cancer incidence is nearly three times higher in countries with higher versus lower HDI (34.9 vs. 13.1 per 100,000), yet mortality is comparable (7.4 vs. 6.9); breast cancer incidence is substantially higher in higher HDI settings (54.1 vs. 33.6), yet mortality is higher in lower HDI countries (14.7 vs. 11.7).^1^ These gaps reflect differences in survival and access rather than in disease biology, and interpreting them requires data on stage at diagnosis, treatment, and timeliness of care that most registries do not collect. A recent global survey indicated that fewer than two thirds of PBCRs publish survival results, and even fewer capture stage, treatment, biomarkers, or socioeconomic factors, with the gap most pronounced in low‐income to middle‐income countries (LMICs).^5^

## OVARIAN CANCER: A CASE STUDY IN DATA COMPLEXITY

If the limitations described by Sung and colleagues are felt across all cancers, they are magnified for malignancies that are inherently difficult to diagnose, classify, and treat. Epithelial ovarian cancer is a clear example. In global cancer statistics 2024, it ranks only 18th overall in incidence but accounts for a disproportionate share of cancer deaths among women, with roughly 330,000 new cases and 203,000 deaths estimated for 2024. This is one of the highest mortality‐to‐incidence ratios of any solid tumor.^1^, ^6^ Its diagnostic, histologic, and management complexity helps explain why the accuracy of the report is so difficult to achieve.

No effective screening test exists; approximately 80% of patients present with advanced‐stage disease, often after months of nonspecific symptoms, and the *front door* to initial diagnostic assessment varies considerably across health systems within and across the globe.^6^ Variation in diagnostic approach produces variation in what registries can record.

Epithelial ovarian cancer is not just one disease but, rather, several distinct histologic and increasingly molecular subtypes, the distribution of which vary across the world. This matters because these different subtypes have distinct molecular profiles, origins, and prognoses. Reclassification of ovarian cancers as those originating in the fallopian tube has produced apparent declines in ovarian cancer incidence in some registries, but not all. This has added to some confusion about incidence trends. In addition, subtypes such as clear cell and mucinous carcinoma vary in geographic and ethnic prevalence. Clear cell carcinoma accounts for a substantially larger share of cases in East Asia than in Western populations, yet most registries do not reliably distinguish the subtypes.^6^ Central expert pathology review in clinical trials confirms only a minority of locally diagnosed mucinous ovarian cancers, demonstrating how misclassification corrupts both incidence and survival statistics at the source.

Perhaps the largest area of data complexity is related to cancer management and documentation thereof. Optimal care of patients with ovarian cancer requires specialized surgery, molecular testing for *BRCA1/BRCA2* and homologous recombination deficiency status, and an increasing milieu of immunohistochemical markers as well as access to maintenance therapy, all of which are scarce in most LMICs.^6^ The absence of histology‐specific natural‐history data leaves regulatory thresholds for new therapies in rare subtypes poorly defined and difficult to contextualize without reliable baseline statistics.

These data gaps align with the survival disparities that global cancer statistics 2024 reports. The report shows that the cumulative risk of developing cancer rises steeply with HDI while the risk of dying does not, a pattern that reflects differential access to early detection and treatment as much as differential exposure but not differences in inherent biology.^1^ The SURVCAN‐3 benchmarking study (The Cancer Survival in Africa, Asia, and South America project) identified *3‐year survival gaps of 20–50 percentage points between low and very high HDI settings* across many cancers, including ovarian cancer, with relative equity only among uniformly poor‐prognosis cancers.^7^ Because Sung and colleagues project the steepest relative increases in lower HDI countries, and because those settings have the least complete registration data, the coming rise in cases is likely to include a disproportionate share of subtypes that are currently under‐registered and thus under‐recognized. This represents a data gap that global statistics are not yet equipped to capture but are critical to setting policy and evaluating capacity for appropriate care provisions on a population level.

The VENUSCANCER project is an example of a more robust registry and how this can drive more data‐based policy decisions. VENUSCANCER analyzed individual records from more than 275,000 women across 39 countries and documented wide variation in the time from diagnosis to treatment, with the median interval from epithelial ovarian cancer diagnosis to surgery exceeding 4 months in some LMICs compared with under 1 month in high‐income settings.^8^ Timeliness metrics of this kind, which most registries do not collect, enable the incidence and mortality counts in global cancer statistics 2024 to be translated into capacity planning.

## STRENGTHENING THE DATA INFRASTRUCTURE

The global cancer statistics report is at its most reliable when it draws on mature registry collaborations, including the European Network of Cancer Registries, China's National Cancer Registry Program, the African Cancer Registry Network, and the SURVCAN survival database.^1^ Extending it beyond these rich and well‐established programs requires sustained investment in PBCRs, particularly in LMICs. Population‐level cancer registration has been estimated to cost only 1–2 US cents per person in several lower income countries. Yet approximately only 25% of low‐income countries have PBCR data compared with the majority of high‐income countries.^3^, ^9^ Registries also need to expand beyond basic incidence and mortality to capture stage, treatment, histologic subtype, biomarker and molecular results, and socioeconomic and comorbidity data, supported by investment in the pathologists, sonographers, and data managers on whom both accurate diagnosis and accurate data depend.^3^, ^5^

Harmonization of methods between GLOBOCAN and the Global Burden of Disease study, where feasible, would reduce confusion among policymakers while preserving the independent cross checking that methodological diversity provides. Regulatory benchmarks for rare subtypes, in parallel, should encourage cross‐group, multinational collaboration and preplanned analyses across histologies, an agenda central to the recently launched Lancet Commission on Ovarian Cancer, which aims to draw new data from multiple registries and databases to drive equity‐focused reform.^10^

## CONCLUSION

Global cancer statistics 2024 provides critically important benchmark data, with clear caveats regarding where heterogeneity of the data affects interpretation. Accurate measurement of the cancer burden requires complete registration, reliable mortality data, and meaningful clinical annotation; without these, the opportunities identified in the report will be missed. An estimated 48% of cancer deaths worldwide are potentially avoidable through prevention, early detection, and improved treatment access; however, realizing that potential depends on knowing where to consolidate resources and target policy changes.^1^, ^11^ As the global burden climbs toward 31.5 million cases by 2050, strengthening the data infrastructure that underpins reports like this one remains among the most cost‐effective investments the cancer community can make.^1^, ^3^

## CONFLICT OF INTEREST STATEMENT

Christina R. Washington reports personal/consulting or advisory fees from AbbVie Inc., EISAI Inc., Foundation Medicine Inc., Karyopharm Therapeutics Inc., and Merck and Company Inc. outside the submitted work. Kathleen N. Moore reports grants/contracts from GlaxoSmithKline LLC and Merck; and personal/consulting or advisory fees from AADI, AbbVie, AstraZeneca, BioNTech, CARIS, Corcept Therapeutics, Daiichi‐Sankyo Inc., Duality Biologics, EISAI Inc., Eli Lilly and Company, Genmab, Gilead Sciences Inc., GlaxoSmithKline, ImmunoGen, Incyte Corporation, MacroGenics, Merck, Mersana, Pfizer, Regeneron Pharmaceuticals, Inc., Schrodinger, Seagen Inc., Takeda Oncology, Verastem, Inc., and Zentalis Pharmaceuticals outside the submitted work. Isabelle L. Ray‐Coquard reports grants/contracts from Genentech; and personal/consulting fees from AbbVie, Amgen, AstraZeneca, Clovis Oncology Inc., Daiichi‐Sankyo Company, DECIPHERA, EISAI Inc., F. Hoffmann‐La Roche, GlaxoSmithKline, ImmunoGen, Mersana, Novartis Pharma, ONCXERNA, PharmaMar, and TESARO Inc. outside the submitted work.
